# Unveiling the dual nature of *Lactobacillus*: from cariogenic threat to probiotic protector—a critical review with bibliometric analysis

**DOI:** 10.3389/froh.2025.1535233

**Published:** 2025-01-31

**Authors:** Di Fu, Xingyue Shu, Lin Yao, Ge Zhou, Mengzhen Ji, Ga Liao, Yunwo Zhu, Ling Zou

**Affiliations:** ^1^ State Key Laboratory of Oral Diseases, National Center for Stomatology, National Clinical Research Center for Oral Diseases, Sichuan University, Chengdu, Sichuan, China; ^2^ Department of Information Management, Department of Stomatology Informatics, West China Hospital of Stomatology, Sichuan University, Chengdu, China; ^3^ State Key Laboratory of Oral Diseases, National Center for Stomatology, National Clinical Research Center for Oral Diseases, Department of Geriatric Dentistry, West China Hospital of Stomatology, Sichuan University, Chengdu, Sichuan, China; ^4^ State Key Laboratory of Oral Diseases, National Center for Stomatology, National Clinical Research Center for Oral Diseases, Department of Conservative Dentistry and Endodontics, West China Hospital of Stomatology, Sichuan University, Chengdu, Sichuan, China

**Keywords:** lactobacilli, dental caries, cariogenicity, probiotics, caries prevention, early childhood caries, bibliometrics

## Abstract

**Introduction:**

Dental caries is a prevalent oral disease with a multifactorial etiology. *Lactobacillus* has been implicated in caries progression on account of its acidogenic properties; On the other hand, they constitute one of the potential probiotic strategies for preventing dental caries. This complex relationship renders the relationship between *Lactobacillus* and dental caries remains ambiguous.

**Methods:**

The Web of Science core collections (WoSCC) were searched to acquire articles relevant to *Lactobacillus* and dental caries. After retrieval and manual screening, publications were analyzed by VOSviewer.

**Results:**

Sweden, the US, and China, which have been the center of international cooperation, have produced the most publications in the research area. Caries Research is the main counterpart journal in the field. “Dental caries”, “*Streptococcus mutans*”, “Lactobacilli”, “Probiotics”, and “Children” have been commonly used as keywords.

**Discussion:**

Based on bibliometric analysis, this study reviews the relationship between lactobacilli and dental caries, emphasizing their dual roles. The detection rate of lactobacilli is closely associated with the incidence and severity of dental caries. However, under specific environmental conditions, these bacteria also exhibit potential probiotic properties that may aid in the prevention of dental caries. Additionally, *Lactobacillu*s is strongly associated with early childhood caries, a specific type of caries.

## 1 Introduction

As a prevalent oral disease with a multifactorial etiology, dental caries have become a significant burden on the public health prevention system (1, 2). According to the World Health Organization, it is estimated that approximately 2.3 billion people worldwide are afflicted with dental caries of permanent teeth and 530 million children have caries of primary teeth (3, 4). Dental plaque refers to the bacterial biofilm attached to the surface of teeth, which is one of the causes of caries and one of the main goals of caries prevention and treatment.

It is postulated that the bacteria of the genus *Lactobacillus* play a crucial role in the further development of caries, particularly in dentin (5). They have consistently been identified at caries sites, whether in the past through traditional clinical isolation and cultivation technology (6, 7), later with 16S sequence analysis (8, 9), or more recently with deep sequencing (10). In recent years, research on *Lactobacillus* has shifted from its cariogenicity to the potential of anti-cariogenic probiotics. They constitute one of the potential probiotic approaches for the prevention of dental caries, which has been demonstrated both *in vitro* (11) and in clinical studies (12).

This complex relationship renders the study of *Lactobacillus* and dental caries less clear than that of *Streptococcus mutans*. The pathogenic mechanisms and specific contributions of lactobacilli are still subjects of debate. Against this backdrop, conducting a review of existing literature to elucidate the current state of research, and identifying hot topics is essential. This review aims to comprehensively analyze the research landscape of *Lactobacillus* in the context of dental caries, emphasizing its pathogenesis, epidemiology, and potential therapeutic applications. By critically evaluating existing findings, the review seeks to identify knowledge gaps, inspire innovative experimental approaches, and optimize research resource allocation.

Bibliometrics, a crucial research methodology, provides a comprehensive and systematic perspective through the analysis of the quantity and trends within a considerable body of relevant literature (13). Via statistical and quantitative analysis, bibliometrics facilitates the identification of trending topics, clarification of the trajectory of disciplinary development, and provision of crucial reference points for the academic community and decision-makers (14). In the field of dentistry, bibliometrics has been applied to research areas such as root caries (15), orthodontics (16), and oral oncology (17). To the best of our knowledge, there is currently no bibliometric analysis conducted in the research field related to lactobacilli and dental caries.

## 2 Materials and methods

### 2.1 Search strategy

The search was performed on March 18, 2024, on the Web of Science Core Collections (WoSCC) database. When performing a bibliometric analysis, WoSCC is one of the most popular scientific source databases for the superiority of supplying data for reference analysis. The retrieval was Topic = (dental caries) AND Title = (Lactobacilli OR Lactobacillus). The inclusion criteria for this analysis were original research articles that explored the role of *Lactobacillus* in dental caries, either in terms of prevention or progression. Exclusion criteria included studies that did not involve *Lactobacillus* or dental caries, those that did not report caries-related outcomes, and studies that were non-peer-reviewed or just abstracts or reviews. The full record and cited references of the retrieved articles were exported in plain text format for subsequent analyses.

### 2.2 Data selection strategy

The article selection process followed a previously reported methodology (18). In brief, two independent reviewers (D.F and L.Y) conducted an autonomous screening of titles and abstracts, and when required, evaluated full texts to identify pertinent studies. Disagreements between the two reviewers were resolved through discussions facilitated by a third reviewer with substantial experience (L.Z).

### 2.3 Data analysis tool

The final included literature was exported and analyzed using the professional bibliometric analysis software VOSviewer, owing to its capability to generate clear and easily interpretable visualizations (19).

## 3 Results

### 3.1 Number of publications

**Figure 1:**
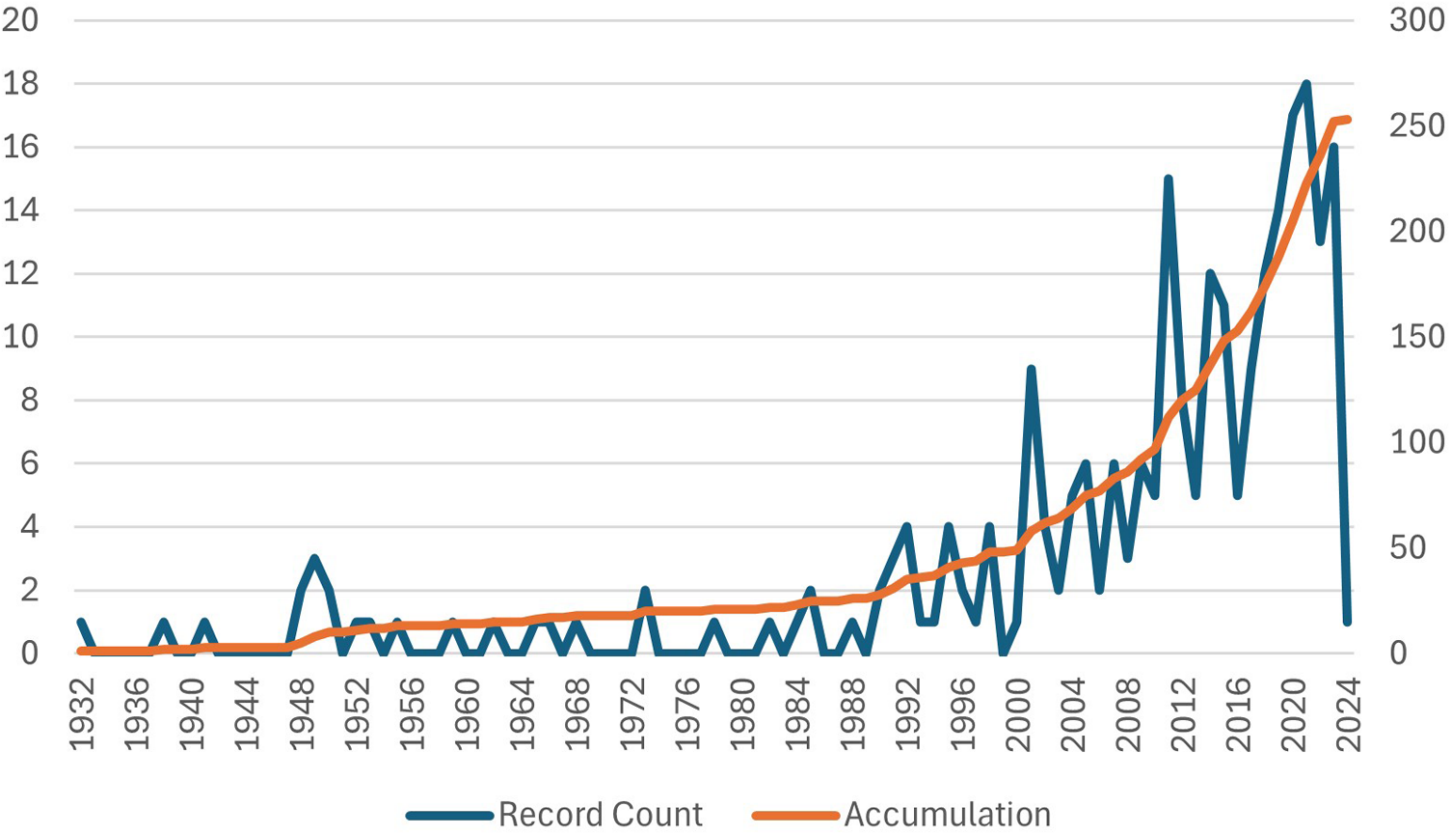
Number of publications relevant to *Lactobacillus* and dental caries.

A total of 274 articles related to *Lactobacillus* and dental caries were retrieved. After screening, 21 articles were excluded (unrelated to dental caries = 16, non-article = 5). A total of 253 articles on *Lactobacillus* and dental caries were published and indexed in WoSCC, and the number of publications was generally on the increase (Figure 1).

### 3.2 The analysis of countries

**Figure 2:**
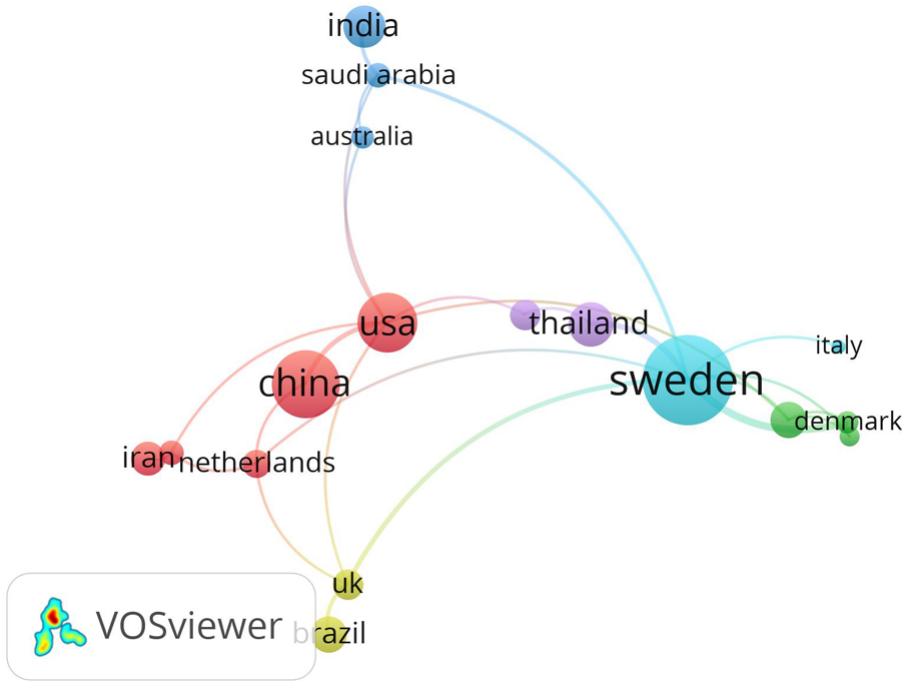
The cooperative network of countries in the *Lactobacillus* and dental caries research area.

Table 1 shows the top 10 most productive countries in the *Lactobacillus* and dental caries research area. Sweden emerged as the country with the highest publications (*n* = 34), followed by China (24) and the USA (22). Figure 2 illustrates the collaboration among 17 countries with more than 4 publications. Each node in the graph represents a country. Nodes of the same color indicate countries belonging to the same cluster. The lines between nodes represent collaborative relationships between the corresponding countries.

**Table 1:** The top 10 productive countries and institutions.

Countries	Record count	Affiliation	Record count
Sweden	34	University of Gothenburg	16
China	24	Prince of Songkla University	15
USA	22	University of Turku	11
Thailand	16	Umea University	9
India	15	University of Copenhagen	8
Brazil	13	University of London	8
Finland	13	University of Amsterdam	5
Iran	12	Tanbah University	5
South Korea	12	University of Sydney	5
UK	11	Egyptian Knowledge Bank	4

Sweden, as the leading contributor in terms of publications, focuses its research primarily on two key areas: (1) the potential anti-caries properties of *Lactobacillus* as probiotics, including their effects on cariogenic pathogens such as *S. mutans* (20, 21) and their preventive efficacy in various populations, such as orthodontic patients, children with early caries lesions, high-caries-risk schoolchildren, and elderly individuals with root caries (22–25); (2) the load, and genotypes of *Lactobacillus* species in the oral environment or carious sites (26–29). The United States, the second-largest contributor to the publications, has placed particular emphasis on the interactions between various *Lactobacillus* species and other oral microorganisms, such as *S. mutans*, *Candida albicans* biofilms, and even multispecies biofilms (30–35).

### 3.3 The analysis of institutions

Table 1 includes the top 10 institutions with the largest number of publications, with the University of Gothenburg being the only institution having more than 15 publications followed by Prince of Songkla University (15) and University of Turku (11).

### 3.4 The analysis of authors

The top 10 most productive authors are shown in Table 2. Teanpaisan R (15) emerge as the author with the highest publications, followed by Piwat S (11) and Twetman S (10). Besides, Twetman S acquires the highest 51 h-index. Teanpaisan R and Piwat S are affiliated with the same research institution, Prince Songkla University. Their research primarily focuses on the probiotic potential of *Lactobacillus*, particularly strains like *L. rhamnosus SD11* and *L. paracasei SD1* (36–41). Twetman's work has involved the use of *L. reuteri* and *L. rhamnosus* in the prevention of caries (23, 42, 43). In addition, his research has explored the potential of probiotics to bind fluoride (25, 44).

**Table 2:** The top 10 productive authors.

Author	Affiliation	H-index	Record count
Teanpaisan, Rawee	Prince of Songkla University	26	15
Piwat, Supatcharin	Prince of Songkla University	17	11
Twetman, Svante	University of Copenhagen	51	10
Soderling, Eva	University of Turku	30	7
Tenovuo, Jorma	University of Turku	44	5
Pahumunto, Nuntiya	Prince of Songkla University	10	4
Kohler, Brady	University of Nebraska Lincoln	33	4
Xiao, Jin	University of Rochester	12	4
Stecksen-Blicks, Christina	Umea University	22	4
Beighton, David	King's College London	52	4

### 3.5 The analysis of journals

A total of 133 journals in the WoSCC database published studies relevant to *Lactobacillus* and dental caries. Table 3 shows the top 10 journals by the number of publications. Caries Research is the number one source with 24 articles, followed by Archives of Oral Biology with 20 articles and Journal of Dental Research with 10 articles.

**Table 3:** The top 10 productive journals.

Journal	IF	Record count
Caries Research	4.2	24
Archives of Oral Biology	3	20
Journal of Dental Research	7.6	10
Acta Odontologica Scandinavica	2	8
Oral Microbiology and Immunology	2.807	8
Clinical Oral Investigations	3.4	6
Community Dentistry and Oral Epidemiology	2.3	6
Journal of Dental Sciences	3.5	5
Microbial Pathogenesis	3.8	4
Anaerobe	2.3	3

### 3.6 The analysis of keywords

**Figure 3:**
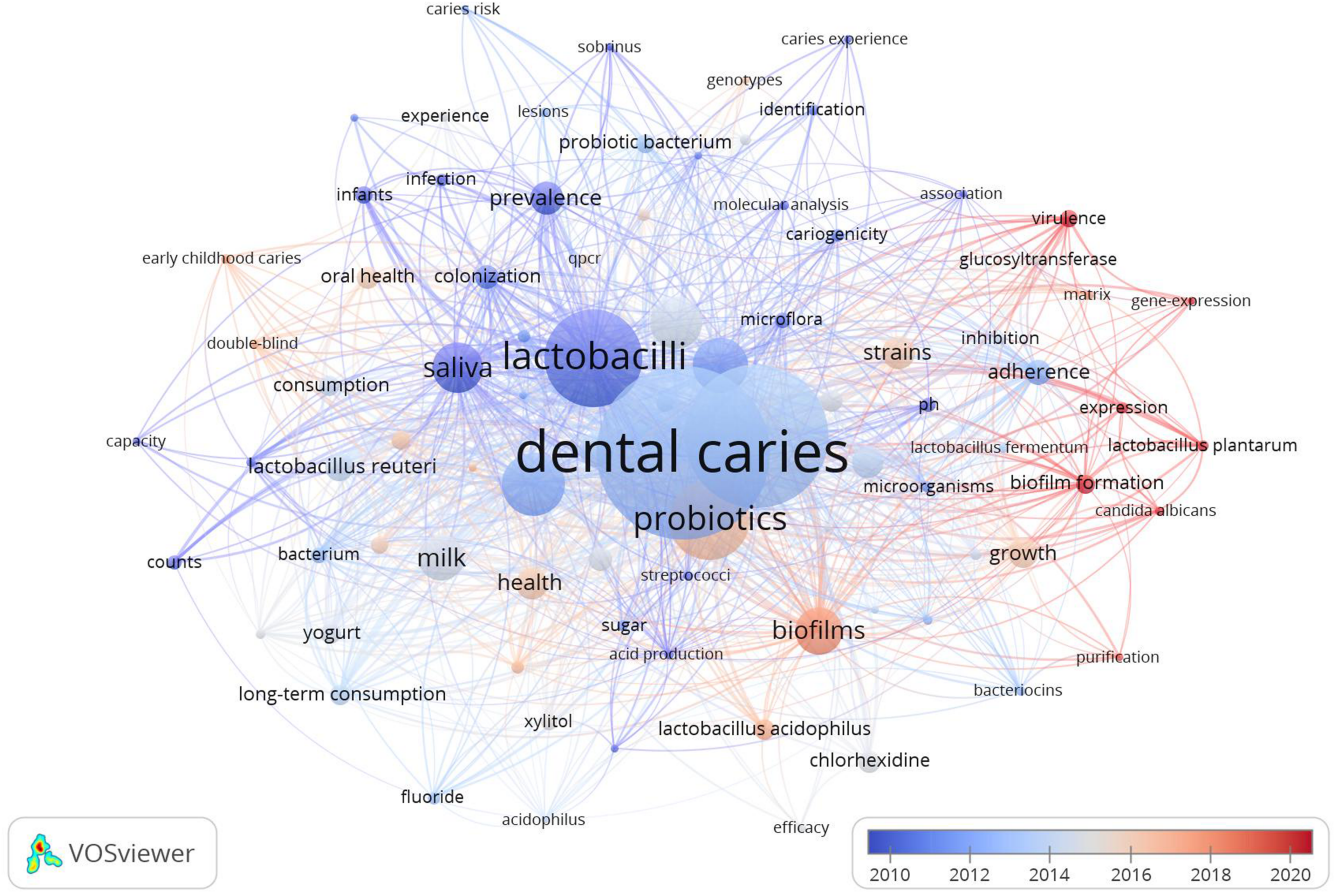
The co-occurrence analysis of keywords in the *Lactobacillus* and dental caries research area.

The top 20 occurrent keywords are listed in Table 4. The “dental caries”, “*Streptococcus mutans*”, “lactobacilli”, “probiotics”, and “children” are the most used keywords. In VOSviewer, setting the minimum of occurrences of a keyword as 8, 45 keywords that met the requirement were used to depict the co-occurrence map (Figure 3). Nodes of the color correspond to the average time of the keywords appear. The lines between nodes represent the occurrence relationship. Table 4; Figure 3 shows the hot topics in the field of *Lactobacillus* and dental caries. The keywords “colonization,” “adherence,” “virulence,” and “pH” highlight a significant research focus on the pathogenic mechanisms of *Lactobacillus* in dental caries, while terms like “prevalence,” “strains,” and “risk” underscore efforts to elucidate the relationship between the abundance and distribution of *Lactobacillus* and the epidemiological characteristics of dental caries; (2) The frequent mention of the keyword “probiotics”, “prevention” and “milk” suggests a growing interest in exploring the potential probiotic effects of *Lactobacillus* in caries prevention and management; (3) early childhood caries (ECC).

**Table 4:** The top 20 occurrent keywords.

Keyword	Occurrence	Keyword	Occurrence
Dental caries	174	lactobacillus rhamnosus	25
Streptococcus mutans	139	prevalence	24
Lactobacilli	87	strains	24
Probiotics	69	health	23
Children	51	*in vitro*	23
Dental plaque	44	growth	21
Bacteria	41	lactobacillus reuteri	20
Saliva	40	adherence	17
Biofilms	36	colonization	16
Milk	35	prevention	16

### 3.7 The analysis of highly cited articles

Information on the top ten highly cited articles is presented in Table 5. The most frequently cited article, with 376 citations, was a long-term clinical follow-up study of *L. rhamnoses* used in children with caries or high caries risk (45). The second most cited article, with 269 citations, was a study published in 2004 by Byun et al., which explored the genetic diversity of lactobacilli at advanced dental caries based on 16S ribosomal DNA technology (8).

**Table 5:** The details of the top 10 highest-cited articles.

First author	Title	Year	Cited time
Näse, L	Effect of long-term consumption of a probiotic bacterium, Lactobacillus rhamnosus GG, in milk on dental caries and caries risk in children.	2001	376
Byun, R	Quantitative analysis of diverse Lactobacillus species present in advanced dental caries.	2004	269
IKEDA, T	Changes in streptococcus-mutans and lactobacilli in plaque in relation to initiation of dental-caries in negro children.	1973	178
Nikawa, H	Lactobacillus reuteri in bovine milk fermented decreases the oral carriage of mutans streptococci.	2004	146
Wasfi, R	Probiotic Lactobacillus sp inhibit growth, biofilm formation and gene expression of caries-inducing Streptococcus mutans.	2018	143
Krüger, C	In situ delivery of passive immunity by lactobacilli producing single-chain antibodies.	2002	143
KOHLER, B	The effect of caries-preventive measures in mothers on dental-caries and the oral presence of the bacteria streptococcus-mutans and lactobacilli in their children.	1984	130
Stecksén-Blicks, C	Effect of Long-Term Consumption of Milk Supplemented with Probiotic Lactobacilli and Fluoride on Dental Caries and General Health in Preschool Children: A Cluster-Randomized Study.	2009	125
Çaglar, E	Effect of chewing gums containing xylitol or probiotic bacteria on salivary mutans streptococci and lactobacilli.	2007	97
Simark-Mattsson, C	Lactobacillus-mediated interference of mutans streptococci in caries-free vs. caries-active subjects.	2007	90

## 4 Discussion

To the best of our knowledge, this is the inaugural study to apply bibliometric analysis to the research domains of *Lactobacillus* and dental caries. Our review analyzes the relationship between lactobacilli and dental caries through the lens of visualized bibliometrics, evaluating the current state and future directions of this field based on empirical evidence rather than subjective opinions.

The increasing number of publications on the relationship between dental caries and lactobacilli over the years indicates a growing interest in this topic, suggesting that it is emerging as a significant research focus. The rising importance of dental caries prevention in clinical practice and public health may stimulate the research area. Cooperation between countries in *Lactobacillus* and dental caries research area predominantly concentrated among a few scientifically advanced nations, such as the United States, Sweden, which occupy central positions in the collaboration network. Authors such as Teanpaisan R, Piwat S, and Twetman S, with a high yield, may be leading figures in the research field of lactobacilli and dental caries, playing a significant role in driving progress and fostering innovation in this domain.

**Figure 4:**
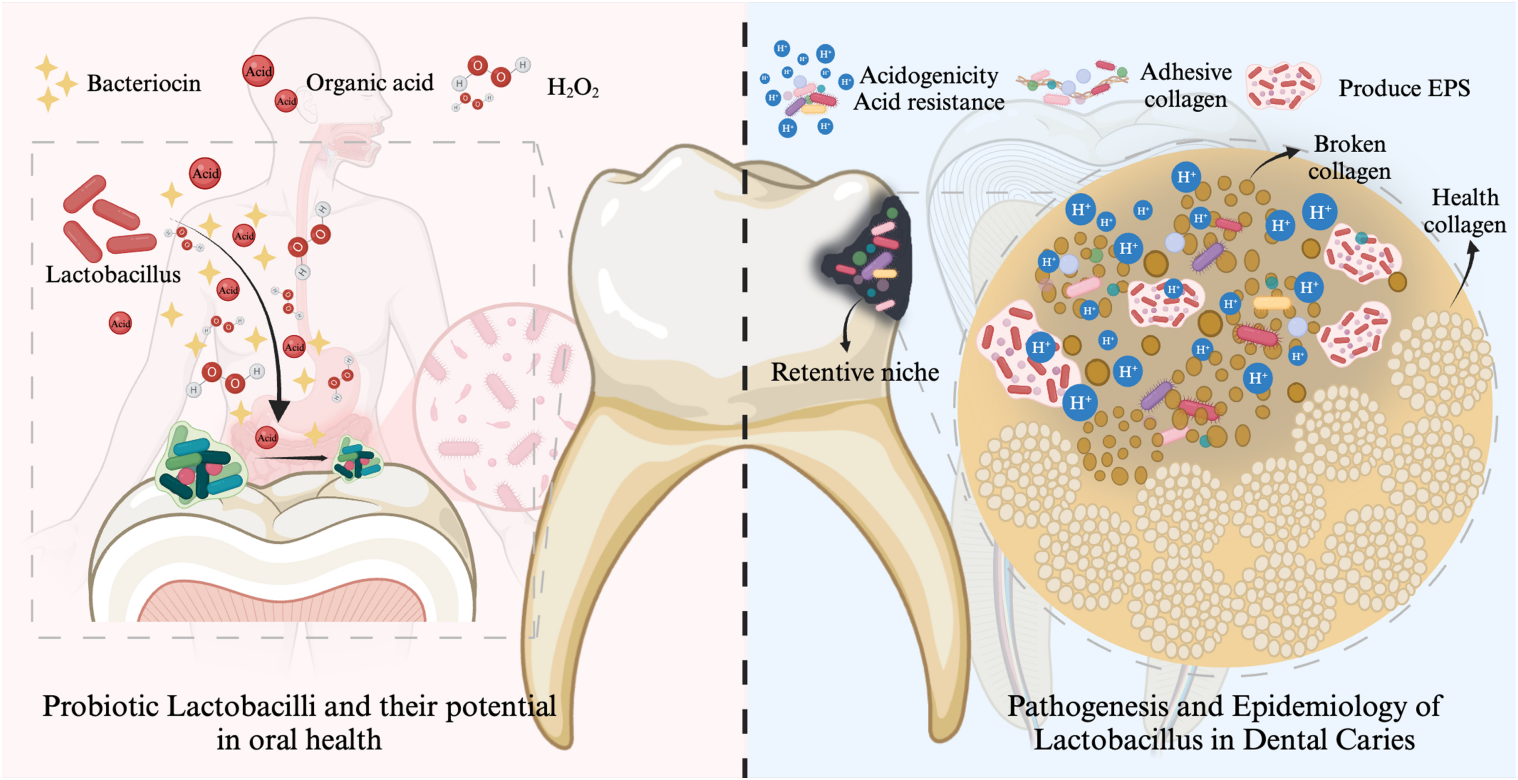
Major research hotspots in the field of *Lactobacillus* and dental caries. The cariogenic potential of *Lactobacillus* is primarily attributed to its acidogenicity, aciduricity, collagen adhesion properties, and biofilm-forming capacity. However, certain *Lactobacillus* strains may exhibit protective effects against dental caries by producing bacteriocins, H₂O₂, and organic acids, facilitating competitive adhesion and colonization, inhibiting cariogenic biofilms, and modulating the host immune response. Created with Biorender.com.

As mentioned in the results, the hot topics in the field of *Lactobacillus* and dental caries include the following three (Figure 4).

### 4.1 Pathogenesis and epidemiology of *Lactobacillus* in dental caries

The cariogenic virulence of *Lactobacillus* has been summarized in the literature (46–48). The cariogenic potential of *Lactobacillus* is primarily attributed to its acidogenicity, aciduricity, adhesion collagen properties, and biofilm-forming capacity (Figure 4). Lactobacilli possess a diverse array of enzymes, including glycoside hydrolases, glycosyltransferases, and isomerases, which enable them to metabolize a wide range of carbohydrates, particularly the oligosaccharides and starches abundant in the oral cavity, resulting in acid production (46, 49). Various *Lactobacillus* species, including *L. plantarum*, *L.salivarius*, *L. rhamnosus*, and *L. casei/paracasei*, have demonstrated the ability to reduce the pH to the critical threshold for enamel demineralization (pH 5.5) within 2.5–3 h (50). *Lactobacillus* species have evolved complex physiological and molecular mechanisms to survive and adapt to acid stress. These mechanisms include modifications to the cell membrane, activation of ATPase proton pumps, metabolic regulation, and the production of macromolecular protective and repair proteins (51). *Lactobacillus* species exhibit relatively limited biofilm formation capabilities and weak adhesion to healthy tooth surfaces (31, 52, 53). However, it is noteworthy that when *S. mutans* and/or other early colonizers establish retention sites, the biofilm formation of *Lactobacillus* significantly increases (31, 54).

From an epidemiological point of view, the abundance and diversity of *Lactobacillus* are closely related to caries risk. Based on previous literature (46, 54, 55), we further summarized the species of *Lactobacillus* detected at caries sites (Table 6). It can be seen that the types of *Lactobacillus* detected in different populations/individuals are different.

**Table 6:** Species of lactobacilli identified in caries.

Identified species	Ozaki et al.	Botha et al.	Martin et al.	Buyn et al.	Munson et al.	Chhour et al.	Schüpbach et al.	Hahn et al.	Chavez de Paz et al.	Obata et al.	Kianoush et al.	Nancy et al.	Marchant et al.	Becker et al.
	Adult											Children		
*L. rhamnosus*	NI	+	+	+	+	+	+	+	+		+	++	24%	
*L.fermentum*	NI	+	+	+	+	+	+	+	−	+	+	+	19%	+
*L. casei*	+	+	−	+	+	+	−	+	+			−	38%	
*L. gasseri*	NI	−	−	+	+	+	+	−	−	+				
*L. salivarius*	NI	−	NI	+	+	+	−	−	+	+		−	7%	
*L. paracasei*	NI	+	+	+	−	NI	−	−	+	+				
*L. plantarum*	+	-	+	-	+	NI	−	+	+			+	NI	
*L. crispatus*	NI	−	−	+	−	+	NI	−	+	+	+			
*L. acidophilus*	NI	+	+	−	−	NI	−	+	+			+	NI	
*L. delbrueckii*	NI	−	−	+	+	+	NI	−	+			+	NI	
*L. cellobiosus*		−	−	+	−	−	−					+	34%	

NI, non identifiable with the method used; −, non recovered.

Although *Lactobacillus* is frequently detected in the mouths of children with dental caries, it has not yet been definitively proven that the high detection rate of *Lactobacillus* directly leads to the development of caries. Some views suggest that *Lactobacillus* does not possess the conditions necessary to cause caries. On the contrary, the high detection rate may be more of a passive result of *Lactobacillus* being retained at the site of the lesion after caries develops (53, 54, 56). Future research should further explore the causal relationship between *Lactobacillus* and caries, considering its potential multiple mechanisms of action.

### 4.2 *Lactobacillus* has the probiotic potential of anti-caries

In recent years, probiotics have gained increasing attention in the prevention and treatment of dental caries biofilms. Numerous *in vitro*, animal, and clinical studies have demonstrated the significant potential of *Lactobacillus* in anti-caries activity. We have summarized these studies in Table 7 for the reader's reference. Certain *Lactobacillus* strains might demonstrate protective effects against caries through the production of bacteriocin (21, 32, 70), hydrogen peroxide (H_2_O_2_) and organic acid (71), competitive adhesion and colonization (72), inhibition of cariogenic biofilms (32, 73), and regulation of the host immune system (32, 41, 74) (Figure 4).

**Table 7:** Studies related to the anti-caries effect of probiotics *Lactobacillus.*

Type	Author, year	Species	Study object	Main conclusions
*in vitro*	Wu et al. 2015 (11)	*L. salivarius*	*Sm*	*L. salivarius K35 and K43* effectively suppressed biofilm formation by decreasing both the population of *Sm* and the synthesis of exopolysaccharides.
	Wasfi et al. 2018 (32)	*L. casei*, *L. reuteri*, *L. plantarum, L. salivarius*	*Sm*	*Lactobacillus* can prevent tooth decay by restricting the growth and virulence factors of *Sm*.
	Zeng et al. 2022 (33)	*L. rhamnosus*, *L. plantarum*, *L. salivarius*	*Sm, Ca*	*L. plantarum* effectively reduced biofilm formation by interfering with the symbiotic relationship between *Sm* and *Ca*.
	Banakar et al. 2023 (57)	*L. rhamnosus GG*, *L. reuteri*	*Sm*	PMs from LGG and *L. reuteri* suppress the biofilm formation, metabolic activity, and gtfB gene expression of *Sm*.
	Liang et al. 2023 (58)	*L. plantarum*	*Sm*	The antibiofilm effect of *L. plantarum* CFS on *Sm* is the result of a combined action of multiple components, with LA playing a dominant role.
	Zeng et al. 2023 (34)	*L. plantarum*	*Sm, Ca*	*L. plantarum* inhibits clinical isolates of *Sm* and *Ca*, with its antimicrobial peptide plantaricin effectively suppressing their growth.
	Gu et al. 2022 (59)	*L. paragasseri*	*Sm*	Two purified compounds of *L. paracasei*, iminosugars and glycerol, can inhibit the expression of genes related to biofilm synthesis.
	Luan et al. 2022 (60)	*L. curvatus*	*Sm*	*L. curvatus* and *Pediococcus pentosaceus* AC1-2 can form aggregates with *Sm*, effectively preventing its colonization.
	Jung et al. 2022 (61)	*L. rhamnosus*	*Sm*	The combined use of prebiotic collagen peptides and the probiotic *L. rhamnosus* can influence the virulence of *Sm* in cariogenic biofilms.
	Noda et al. 2021 (62)	*L. reuteri*	*Sm*	The presence of *L. reuteri* significantly reduces the glucan adhesiveness produced by *Sm*.
	Jang et al. 2021 (63)	*L. brevis*	*Sm*	*L. brevis* effectively disrupts *Sm* biofilm formation by inhibiting auto-aggregation, cell surface hydrophobicity, and EPS production.
	Srivastava et al. 2020 (35)	*L. Plantarum*	*Sm, Ca*	*L. plantarum* 108 can inhibit the biofilms of *Sm*, *Ca*, as well as their dual-species biofilm.
Animal experiments	Guo et al. 2023 (64)	*L. paracasei ET-22*	*Sm in vitro*, Dental caries in mice	Supplementing drinking water with ET-22 significantly improves dental caries scores in mice, with both live and heat-killed ET-22 having similar effects on the microbial composition of dental plaque biofilms.
	Zhang et al. 2020 (65)	*L. plantarum*	*Sm* and *Ca* in rats, Dental caries in mice	*L. plantarum* significantly reduces the number of *Sm* and *Ca* in the oral cavity of rats and significantly decreases enamel mineral loss.
	Weng et al. 2022 (66)	*L. acidophilus*	*Sm in vitro*, Dental caries *in vivo* rat model	The outer membrane of encapsulated *Lactobacillus* enhances the adhesion of triclosan nanoparticles to *Sm* biofilms, leading to more effective targeted disruption of biofilm formation and the expression of associated virulence factors.
	Zhang et al. 2020 (67)	*L. plantarum K41*	*S. mutans in vitro and in vivo* Dental caries *in vivo* rat model	*L. plantarum K41* effectively inhibits *Sm* growth, biofilm formation, and dental caries *in vivo*.
	Lin and Pan 2014 (68)	*L. paracasei subsp. paracasei NTU 101*	*Sm in vitro, D*ental caries *in vivo* rat model	Continuous administration of NTU 101 lowered the pH of dental biofilms and reduced enamel demineralization.
Experiments on Humans	Näse et al. 2001 (45)	*L. rhamnosus GG*	Children between 1 and 6 years old	Children who received LGG milk had less dental caries and lower *Sm* counts than regular milk.
	Marttinen et al. 2012 (43)	*L. rhamnosus GG, L. reuteri*	Students of the University of Turku	*Lactobacillus* intake reduced *Sm* counts without significantly affecting the acidity of supragingival plaque.
	Staszczyk et al. 2022 (69)	*L. salivarius*	Children between 3 and 6 years old	Two weeks of daily intake of chewable tablets containing heat-inactivated *L. salivarius* can significantly reduce the incidence and prevalence of dental caries in children within one year.
	Pahumunto et al. 2020 (40)	*L. rhamnosus-SD11*	Children aged 13–14 years old	Daily consumption of maltitol-containing *L. reuteri* SD11 fermented milk can reduce *Sm* in saliva, offering beneficial effects on oral health.
	Pahumunto et al. 2019 (41)	*L. paracasei SD1*	Volunteers aged 12–14 years old	*L. paracasei* SD1 has the ability to control *Sm* levels and stimulate the production of sIgA.

*Sm*, *S. mutans*; *Ca*, *C. albicans*; LGG, *L. rhamnosus GG*; PMs, postbiotic mediators; CFS, cell-free supernatant; LA, lactic acid; EPS, exopolysaccharide.

It is important to note that while lactobacilli have been extensively studied and applied in the prevention of dental caries, the individual variability in its probiotic efficacy remains incompletely understood. Individual differences in oral microbiota, dietary habits, and genetic factors significantly influence the efficacy of lactobacilli (75), with notable variability observed across different age groups, regions, and health conditions, posing challenges for its clinical application. Moreover, the long-term safety of lactobacilli remains an unresolved concern. The acidogenic properties of *Lactobacillus* may exacerbate the acidic environment in the oral cavity under certain conditions, thereby promoting the occurrence and progression of dental caries (76). Additionally, the adhesive properties and biofilm-forming ability of *Lactobacillus* may, in some cases, synergize with other cariogenic bacteria, increasing the risk of oral microbial dysbiosis (31, 77). Therefore, although *Lactobacillus* exhibits probiotic effects in promoting oral health, its safety in clinical applications requires further evaluation, particularly in high-risk populations such as individuals with a history of dental caries or those with compromised immune function (78).

### 4.3 *Lactobacillus* and ECC

It is notable that a particular type of caries emerged in the keyword analysis: early childhood caries (ECC). ECC, defined as the existence of one or more decayed teeth that have been extracted or filled in children under the age of 6, is one of the most prevalent diseases worldwide among children of this age group (79). Research on the relationship between ECC and *Lactobacillus* can be divided into two main categories: one group explores the anti-caries effects of *Lactobacillus* on children with ECC, while the other investigates the load of *Lactobacillus* in the oral environment of children with ECC, such as in saliva and dental plaque (Table 8). ECC progresses rapidly and, similar to rampant caries, can progress rapidly to dentin (90). *Lactobacillus* has a high affinity for dentin collagen (91–93). We speculate that this may be the reason why more lactobacilli tend to be detected at ECC lesion sites.

**Table 8:** Research on ECC and *Lactobacillus.*

Topic	Author, year	Subject	Species	Main conclusions
The load and species of Lactobacillus at ECC carious sites.	Leme et al. 2022 (80)	Preschoolers	–	*L. acidophilus* and *L. gasseri* was associated with ECC in children with normal nutritional status.
	Indiani et al. 2020 (81)	Preschoolers with or without ECC	–	*S. mutans* and *Lactobacillus* levels in the mouth and gut are linked to ECC, with obesity influencing this connection.
	Reis et al. 2021 (82)	Children between 2 and 5 years of age with dentinal lesion	–	The abundant presence of *L. casei* species in active dentinal caries lesions indicates their potential link to caries activity, with the wzb gene possibly playing a key role in caries progression.
	Liu et al. 2019 (83)	Children from 3 to 6 years of age with ECC	–	A higher salivary level of LB was observed in children with more severe caries, although the difference was not statistically significant.
	Klinke et al. 2014 (84)	Infants with ECC	–	The removal and restoration of ECC lesions under general anesthesia effectively reduced cariogenic bacteria and yeasts.
	Ramamurthy et al. 2014 (85)	Children aged 2–5 years with or without S-ECC	–	s-ECC is positively associated with higher salivary levels of both MS and LB in preschool children from low socioeconomic backgrounds.
	Mitrakul et al. 2017 (86)	Children with S-ECC and caries-free	–	The levels of *S. mutans*, Lactobacillus, bifidobacterium, and their ratio to total bacteria were notably higher in both initial and mature dental plaques of children with S-ECC.
	Indiani et al. 2020 (81)	Preschoolers with or without ECC	–	The levels of *S. mutans* and the presence of *Lactobacillus* in both the oral cavity and lower gastrointestinal tract were linked to ECC.
Studies on Lactobacillus-Based Therapies for ECC	Staszczyk et al. 2022 (69)	Children aged 3–6 years with or without ECC	*L. salivarius*	Frequent short-term consumption of probiotics may help decrease the incidence of dental caries.
	Hasslöf et al. 2013 (87)	Infants aged 4 months	*L. paracasei F19*	Early intervention with the probiotic *L. paracasei F19* does not have a long-term impact on caries experience.
	Pahumunto et al. 2018 (39)	Children, aged 1.5–5 years old	*L. paracasei SD1*	Consumption of milk powder containing *L. paracasei SD1* led to a reduction in salivary *S. mutans* levels and delayed the development of new caries.
	Zeng et al. 2023 (34)	*S. mutans* and *C. albicans* Clinical isolates from Children with ECC	*L. plantarum 14917*	*L. plantarum 14917* showed significant inhibition against clinical isolates of *S. mutans* and *C. albicans.*
	Krzyściak et al. 2017 (88)	*S. mutans* and *C. albicans* clinical strains, isolated from dental plaque of patients with ECC	*L. salivarius*	*L. salivarius* may secrete intermediates that inhibit the biofilm formation of *S. mutans* and *C. albicans*.
	Plonka et al. 2012 (89)	Infants	–	The period between 6 and 12 months is a critical window during which key factors can be managed to reduce the colonization of LB.

ECC, early childhood caries; S-ECC, severe early childhood caries; LB, lactobacilli; MS, mutans streptococci.

This study is founded on a bibliometric analysis of the included studies, which inherently possesses several limitations. Firstly, there exists the potential for publication bias. Although the utilization of the WoSCC database contributes to ensuring the credibility and authority of the included data, it might also constrain the comprehensiveness of the information. Additionally, the publications selected for the bibliometric analysis were initially identified through pre-searching with a predefined search formula and then manually filtered. However, the process of data exportation and filtering might result in data loss, misidentification of articles, or other problems. Moreover, analysis software VOSviewer still has scope for improvement. For instance, synonymous entities, such as authors, institutions, and keywords, might fail to be precisely identified or discriminated, giving rise to circumstances where the same institution, author, or synonym is not accurately merged. Furthermore, only English-language studies were encompassed within this analysis, which could have resulted in an underrepresentation of research capabilities from non-English-speaking countries.

## Data Availability

The original contributions presented in the study are included in the article/Supplementary Material, further inquiries can be directed to the corresponding authors.
